# Recrystallization of Commercial Carbamazepine Samples—A Strategy to Control Dissolution Variability 

**DOI:** 10.3390/pharmaceutics4010058

**Published:** 2012-01-13

**Authors:** Felicia Flicker, Veronika A. Eberle, Gabriele Betz

**Affiliations:** Industrial Pharmacy Lab, University of Basel, Mülhauserstrasse 51, Basel 4056, Switzerland

**Keywords:** polyvinylpyrrolidone, polymer, intrinsic dissolution, polymorphism, transformation, morphology

## Abstract

Physical properties of commercial carbamazepine (CBZ) samples can significantly influence drug release and thereby jeopardize bioequivalence of the final dosage form. The aim of this study was to reduce variability in commercial CBZ samples by recrystallization. CBZ samples of four different suppliers were recrystallized in ethanol solution containing 1% polyvinylpyrrolidone (PVP). CBZ samples were analyzed by disk intrinsic dissolution rate (DIDR), X-ray powder diffraction (XRPD), differential scanning calorimetry (DSC), and scanning electron microscopy (SEM). Recrystallized CBZ samples showed strongly reduced variability in DIDR compared to the untreated CBZ samples. Moreover, transformation process to CBZ dihydrate was inhibited; no dihydrate crystals were visible on compact surfaces after 8 h intrinsic dissolution measurement. Recrystallized CBZ samples showed no change in polymorphic form, however, particle size and shape was inhomogenous. In binary mixtures with microcrystalline cellulose, recrystallized CBZ samples again showed difference in drug release. This difference was associated with the inhomogenous particle size in the recrystallized CBZ samples. The results show that a controlled grinding step is required after recrystallization. We suggest the recrystallization in presence of 1% PVP followed by a controlled grinding step as a strategy to reduce dissolution variability in commercial CBZ samples.

## 1. Introduction

Pharmaceutical companies are facing a fast growing market of drug suppliers where raw materials can be obtained from suppliers in India and China at lower price. Physicochemical properties of raw materials can vary among drug suppliers with consequence on the drug performance in the final drug formulation. 

CBZ is one example where sample variability among different suppliers effects the dissolution behavior of the tablet formulation. Šehić *et al.* [1] reported different transformation to dihydrate within commercial CBZ samples, although the same polymorphic form was specified. This difference also shows in tablet formulation using Ludipress^®^ as tablet filler. Developing a CBZ tablet formulation for immediate release, Flicker and Betz [2] selected two excipients, crospovidone and hydroxypopyl cellulose, both reported to inhibit transformation. However, tablet hardness, friability, and drug release varied with the CBZ sample. For CBZ, the variability in drug release is closely linked to clinical failures as the bioavailability of CBZ is dissolution controlled and the pharmacological action is within a narrow therapeutic range [3]. There have been numerous reports showing irregular dissolution [4,5,6], bioinequivalence [7,8,9], and clinical failures of CBZ [10]. 

Physicochemical properties of raw materials depend on the method of production. The crystallization step of a drug can be performed with different solvents and additives. They result in different crystal habits and possible solvent inclusion, both leading to different solubility and dissolution behavior [11]. This phenomenon has also been studied on CBZ. Bolourtchian *et al.* [12] compared CBZ samples recrystallized in ethanol and acetone under different conditions. Crystallization from ethanol or acetone results in polyhedral and thin plate-like crystals, respectively. Using a watering-out method needle-shaped crystals are obtained. The recrystallized CBZ samples show improved dissolution rate and compactibility compared to the untreated samples. However, interpreting the XRPD results of the CBZ crytals obtained by the watering-out method, the crystals are most likely of CBZ dihydrate form. The peaks around 5, 9, 12° 2*θ* conform with the XRPD data reported for CBZ dihydrate [13,14]. Very similar recrystallization studies were performed by Mahalaxmi *et al.* [15] who recrystallized CBZ samples in ethanol and acetone at different cooling conditions. The solvents also lead to different crystal habit and different polymorphic form. Fast cooling results in smaller crystals with faster dissolution. Nokhodchi *et al.* [16] studied the effect of the additives PEG 4000, polyvinylpyrrolidone (PVP K30), and Tween 80 (1% w/v) on the recrystallization of CBZ in ethanol solutions. Thus obtained crystals are of different crystal habit but of same polymorphic form (p-monoclinic CBZ, form III). Recrystallization in presence of PVP K30 results in more block-shaped crystals showing higher dissolution rate and improved tensile strength. 

Polymers such as PVP, hydroxypropyl methylcellulose, and hydroxypropylcellulose, when added at only small amounts (1–4% w/v) to the dissolution medium, are reported to inhibit the transformation of anhydrous CBZ to the less soluble CBZ dihydrate [17,18,19]. 

In this study, CBZ samples of four different suppliers were recrystallized in ethanol containing 1% PVP to reduce dissolution variability. The effect of recrystallization on sample variability was investigated by XRPD, DSC, and intrinsic dissolution profiles using the unidirectional dissolution method developed earlier [20]. Recrystallized samples were also tested in binary mixtures with microcrystalline cellulose at 70% drug load. 

## 2. Materials and Methods

Carbamazepine samples of four different suppliers (CBZ A, B, C and D) were recrystallized according to Nokhodchi *et al.* [16]. Each sample (42 g) was dissolved in 910 mL ethanol containing 1% polyvinylpyrrolidone (PVP K30, Kollidon^®^ K30 BASF) at 65 °C. After cooling to room temperature the solutions were placed in the refrigerator (4–8 °C) for 24 h. The crystals were filtered under suction, dried at ambient condition over night and for 72 h over phosphorous pentoxide (0% RH). The crystals were slightly ground with mortar and pistil and kept at room temperature and 43% RH. Recrystallized CBZ samples were obtained at a yield of 53%–70%. 

Microcrystalline cellulose (MCC SANAQ^®^ 102L; Pharmatrans SANAQ AG, Switzerland) was used as a water-insoluble tablet-filler for the binary mixtures. All other chemicals and reagents purchased from commercial sources were of analytical grade. 

### 2.1. Polymorphic Characterization

Polymorphic form of CBZ samples was characterized by X-ray powder diffractometry (XRPD) using a diffractometer (D5000, Siemens, Germany). The powder was filled into special holders and the surface was pressed flat. Operating conditions were Ni filtered Cu-Kα radiation (*λ* = 1.5406 *Å*), 40 kV, and 30 mA. Step was 0.02° 2*θ*, step time 1.0 s, angular scanning speed 1° 2*θ*/min, and angular range between 5° and 40° 2*θ* scale. 

Thermal behavior of all samples was analyzed by differential scanning calorimetry (DSC), using heat flux DSC (4000, PerkinElmer, USA). DSC was calibrated with indium prior to the measurement. A sample of 3–6 mg was accurately weighted into an aluminum pan with holes and scanned between 40 °C and 220 °C at 10 °C/min under dry nitrogen gas purge (20 mL/min). 

True density of all samples was assessed by a gas displacement pycnometer (AccuPyc 1330, Micromeritics, USA). Powder was purged with helium by five repetitive purging cycles and the density was reported as average value. The test was performed in triplicate. 

### 2.2. Morphological Characterization

For morphological characterization scanning electron microscopy (SEM) (ESEM XL 30 FEG, Philips, The Netherlands) was applied at a voltage of 10 kV and magnifications of 100–2000 times. Before analysis, powder was sprinkled on carbon adhesive and then sputtered with platinum. 

### 2.3. Water Activity Measurement

Water activity of all samples was monitored with a digital water activity analyzer (Hygropalm, Rotronic AG, Switzerland) at 23.5 ± 1.5 °C. 

### 2.4. Unidirectional Dissolution Method

#### 2.4.1. Sample Preparation

Recrystallized CBZ samples were mixed with MCC for 10 min in a mixer (Turbula^®^ type T2C, W. Bachofen, Switzerland) and for a further 2 min after adding 1% magnesium stearate (Sandoz, Switzerland) to the mixture. Drug load was 70% and mixtures of 10 g were prepared. 

Flat-faced compacts of 400 mg were produced from recrystallized CBZ and of binary mixtures using Zwick 1478 material tester (Zwick, Germany). Surface area was 0.95 cm^2^ and porosity was 12% for untreated CBZ samples and binary mixtures and 7% for the recrystallized CBZ samples. Compaction speed was at 10 mm/min for pure drug and at 50 mm/min for the binary mixtures. 

#### 2.4.2. Dissolution

To obtain disc intrinsic dissolution rate (DIDR) profiles of the CBZ samples, unidirectional dissolution was performed by a modified USP Apparatus I. The prepared compact of fixed porosity was placed in a sample holder fitting to the rotating unit of the dissolution apparatus (SotaxAT*7smart*, SOTAX AG, Switzerland) and the compact was embedded in melted paraffin wax so only one surface was available to the dissolution media. Unidirectional dissolution method was performed in 500 mL water at 37 ± 0.5 °C and rotation speed was 100 rpm. Drug content of the media was measured for 2 h at predetermined time intervals by UV-VIS spectrophotometer (Lambda 25, PerkinElmer, USA) at 285 nm. Compacts were also analyzed in a longer run of 8 h under the same conditions but in 1000 mL water to keep sink conditions. A longer run is essential to obtain the full DIDR profile, where the transformation behavior to the CBZ dihydrate can be detected and characterized. 

#### 2.4.3. Evaluation of DIDR Profiles

Intrinsic dissolution rate (IDR) was determined by the slope in the DIDR profile prior to the first inflection point, the slope referring to the release of anhydrous CBZ before transforming to the dihydrate. DIDR profiles were analyzed by inflection points as described in earlier work [20]. The full DIDR profile was reported to describe individual transformation behavior of CBZ raw materials using two inflection points. The first inflection point reflects the onset of dihydrate formation and dissolution presenting a gradual change in the dissolution rate. A second inflection point further reflects the onset of a constant dissolution rate, where the transformation process and dissolution reach an equilibrium state. The first inflection point was determined by the intercept of the linear regression in the initial DIDR profile approximated to best fit (R^2^ ≥ 0.999) and the linear regression through all data points of the later stage in the 2 h-DIDR profile. For all statistical comparison, one-way ANOVA followed by Student’s t-test was applied. 

#### 2.4.4. Analysis of Compact Surface

Compact surfaces were analyzed by SEM (see Section on Morphological Characterization). Prior to the analysis the compacts were kept under controlled RH of 43% for a maximum of 24 h. The compacts were then fixed to the sample holder by conductive silver and sputtered with gold. 

### 2.5. Tensile Strength

Tensile strength (*σ*) was calculated by the Equation (1),

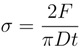
(1)
where *F* is the force needed to fracture the tablet by the hardness tester, *D* the diameter, and *t* the thickness of the cylindrical tablet [21]. Tablet dimensions were measured by a digital caliper and crushing strength by a tablet hardness tester (Dr. Schleuniger^®^ model 8M, Pharmatron, USA). All compacts analyzed were prepared by the method described in Section 2.4. Compacts were of fixed porosity and were kept under controlled condition for elastic recovery over a minimum of 24 h. 

## 3. Results and Discussion

### 3.1. Polymorphic Characterization

**Figure 1:**
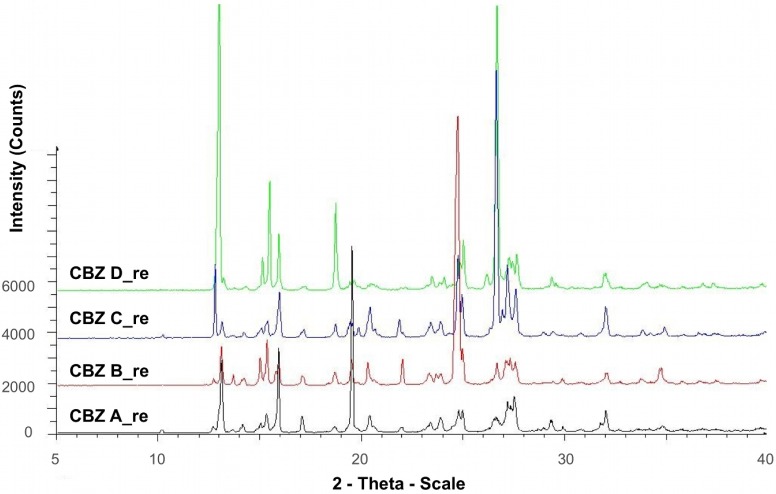
X-ray powder diffraction of recrystallized CBZ samples.

**Figure 2:**
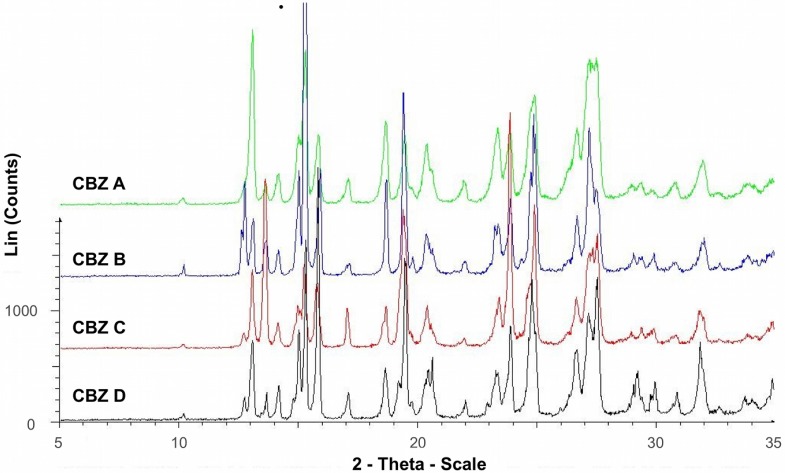
X-ray powder diffraction of untreated CBZ A, B, C and D.

Diffractograms of recrystallized CBZ samples are shown in Figure 1. The diffractograms were consistent with XRPD data of the untreated CBZ samples (Figure 2) and the data reported for CBZ p-monoclinic form, where main peaks show at 2*θ* = 14.9, 15.2, 15.8, 27.2, 27.5, and 32.0° [22]. Peaks at angles smaller than 10° 2*θ* indicating the presence of CBZ triclinic (form I) or trigonal (form II) were not detected in our samples. Moreover, there was no evidence as to the presence of PVP in the recrystallized samples, thus an incorporation of significant amount of PVP into the crystal lattice can be excluded. However, at lower angles of ° 2*θ* diffractograms peak intensities varied, indicating difference in crystallinity, particle size, and preferred orientation. 

**Figure 3:**
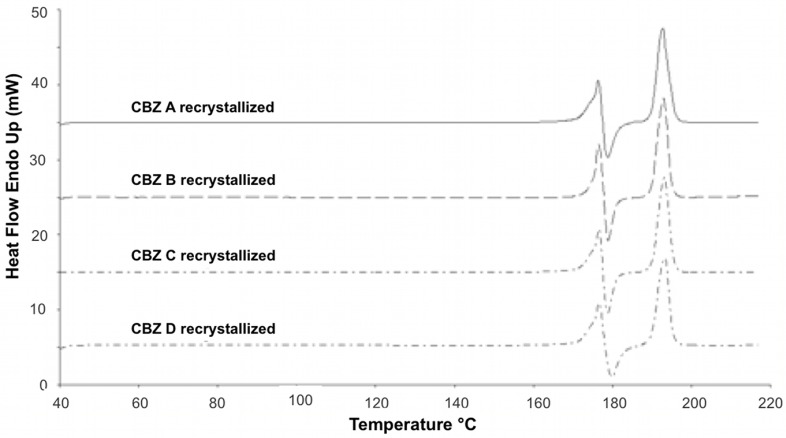
DSC profiles of recrystallized CBZ samples; 10 °C/min scanning rate.

**Figure 4:**
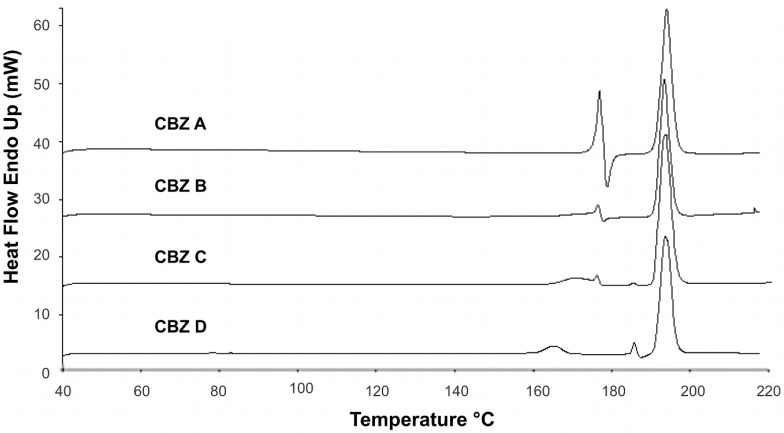
DSC profiles of untreated CBZ samples; 10 °C/min scanning rate.

DSC measurements of recrystallized CBZ showed the characteristic thermal events for CBZ p-monoclinic form (Figure 3) [1,23]. Melting of form III was visible around 176 °C, followed by an exotherm around 182 °C (crystallization of form I). Around 191 °C form I melted. DSC profiles of all recrystallized CBZ samples were all alike. The deviations found in untreated CBZ samples (Figure 4) were removed by the recrystallization. 

True density of all untreated and recrystallized CBZ samples were both 1.340 ± 0.001 g/cm^3^ , indicating CBZ polymorphic form III (p-monoclinic) [23,24]. 

### 3.2. Morphological Characterization

**Figure 5:**
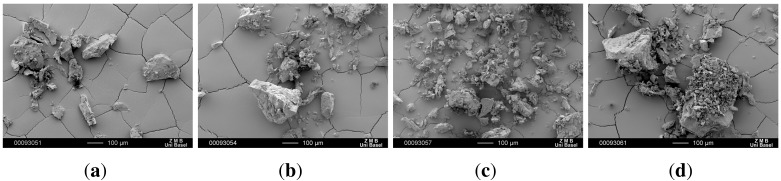
SEM pictures of recrystallized CBZ samples. (**a**) CBZ A re; (**b**) CBZ B re; (**c**) CBZ C re; (**d**) CBZ D re.

SEM images of the recrystallized CBZ samples illustrate inhomogeneous particle size and shape (Figure 5). Crystallization is mostly followed by a milling or grinding step. However, these physical processes cause crystal defects, mechanical activation, and small amounts of amorphous that can further lead to faster and inhomogeneous dissolution behavior of CBZ [25,26,27,28]. CBZ crystals obtained after recrystallization were very big and agglomerated at the bottom of the beaker. To avoid shear forces causing crystal defects the crystal agglomerates were gently broken apart by mortar and pistil prior to the analysis. 

### 3.3. Water Activity

Water activity of recrystallized CBZ samples was 0.327–0.346 and therefore below the critical range for transformation to CBZ dihydrate [29,30]. 

### 3.4. DIDR Profiles of Recrystallized CBZ

**Figure 6:**
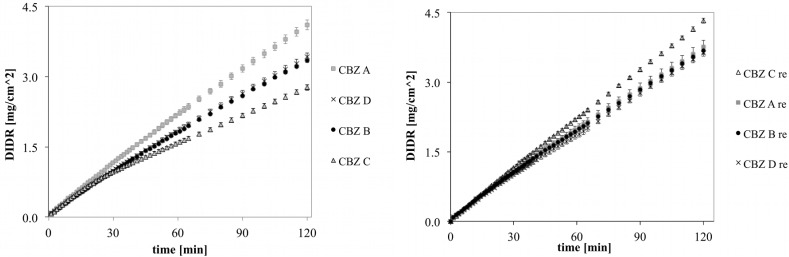
DIDR profiles of untreated (left) and recrystallized (right) CBZ A, B, C and D; 2 h DIDR test. Average values (n ≥ 3) are presented.

**Figure 7:**
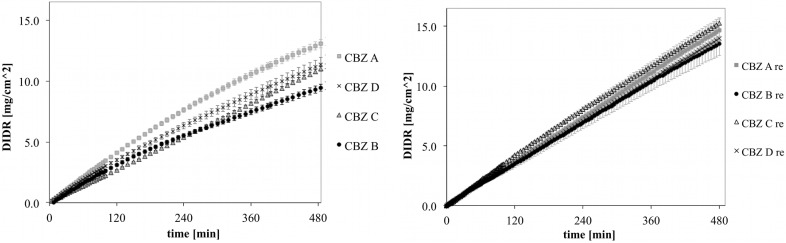
DIDR profiles of untreated (left) and recrystallized (right) CBZ A, B, C and D; 8 h DIDR test. Average values (n ≥ 3) are presented.

DIDR profiles of untreated and recrystallized CBZ A, B, C and D are shown in Figure 6. The variability observed for untreated CBZ samples was clearly reduced for recrystallized CBZ samples. This was confirmed by the longer DIDR test of 8 h (Figure 7). IDR values of recrystallized CBZ were 35.3 ± 2.3 µg/cm^2^/min, slightly lower than the average IDR of untreated CBZ samples and with less variability. The average IDR values of untreated CBZ was 36.6 ± 8.5 µg/cm^2^/min. The variability in IDR of untreated CBZ samples has been associated by difference in morphology as well as small amounts of amorphous and polymorphic impurities [20]. 

Inflection points in DIDR profiles of untreated and recrystallized CBZ samples are shown in Table 1. Untreated CBZ samples could be differed by their inflection points (ANOVA, *p* < 0.005) , which conforms to the earlier findings [20]. In contrast, the inflection points of recrystallized CBZ samples were not distinct (*p* > 0.05). A more robust and standardized CBZ sample presents a major advantage to pharmaceutical companies because dissolution variability in CBZ has been directly linked to clinical failures of the final drug product [10]. 

**Table 1:** Inflection points (IP) in DIDR profiles of untreated and recrystallized CBZ samples.

Sample n = 3	IP untreated [min]	IP recrystallized [min]
CBZ A	35 ± 6	31 ± 10
CBZ B	23 ± 6	28 ± 4
CBZ C	19 ± 2	24 ± 6
CBZ D	26 ± 6	21 ± 3

**Figure 8:**
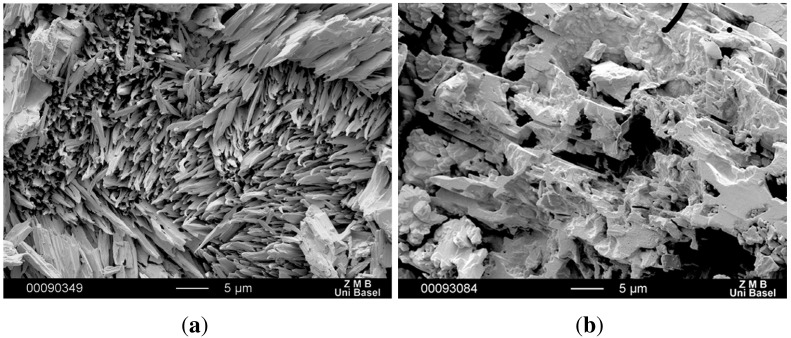
SEM picture of compact surface of untreated CBZ (**a**) and of recrystallized CBZ (**b**) after 8 h DIDR test.

SEM images confirmed the characteristic needle-structure of CBZ dihydrate on the compacts of untreated CBZ samples (Figure 8(a)), which are responsible for the inflection points [20]. It is interesting to note that there was a lack of dihydrate formation on recrystallized CBZ samples. SEM images revealed no dihydrate crystals on the compact surfaces, not even after 8 h of DIDR measurement (Figure 8(b)). The transformation was inhibited and therefore the inflection point for recrystallized CBZ was caused by a change in compact surface not related to the formation of CBZ dihydrate. 

CBZ samples were recrystallized in presence of 1% PVP. The polymer PVP was found to prevent the transformation to the CBZ dihydrate, inhibiting the nucleation and crystal growth if present in the dissolution media at 1% PVP [18,31]. The formation of CBZ dihydrate in dissolution is a surface phenomenon. We assume that during the recrystallization some PVP molecules adsorbed to the crystal surface and formed a protective layer to inhibit dihydrate formation on site. The ability of PVP to adsorb on crystal surfaces has been reported earlier by Wen *et al.* [32]. They detected that recrystallization in presence of PVP partially changes the dissolution pattern of acetaminophen crystals. This change was associated with the adsorption of the polymer PVP on crystal surface by van der Waals interaction. In this study, the amount of PVP adsorbed to the recrystallized samples is expected to be very small as no solubilizing effect was observed. Furthermore, XRPD and DSC results showed no indication of PVP amount in the recrystallized samples. 

### 3.5. Properties of CBZ Compacts

Table 2 shows the tensile strength of the CBZ compacts and the respective compaction forces for untreated and recrystallized CBZ samples. There was a narrow compaction force range for each CBZ sample showing insufficient binding below this range, above lamination and capping occurred. Best compacts were obtained with compaction forces of 6.3–10.5 MPa for untreated CBZ (porosity of 12%) and of 21.0 MPa for recrystallized CBZ samples (porosity of 7%). Compacts of recrystallized CBZ samples did not show any improvement in compaction as expected by the results reported by Nokhodchi *et al.* [16]. Nonetheless, the variability in tensile strength among compacts of recrystallized CBZ was reduced. 

**Table 2:** Compaction force (CF) and tensile strength (TS) of untreated and recrystallized CBZ samples. Compacts were of fixed porosity.

n ≥ 5	untreated CBZ		recrystallized CBZ	
	CF [MPa]	TS [MPa]	CF [MPa]	TS [MPa]
CBZ A	10.5	0.355 ± 0.036	21.0	0.630 ± 0.098
CBZ B	8.4	0.505 ± 0.127	21.0	0.556 ± 0.262
CBZ C	6.3	0.294 ± 0.017	21.0	0.436 ± 0.252
CBZ D	8.4	0.651 ± 0.095	21.0	0.498 ± 0.180
*x* ± SD	8.4	0.451 ± 0.160	21.0	0.530 ± 0.083

### 3.6. Recrystallized CBZ in Binary Mixtures

**Figure 9:**
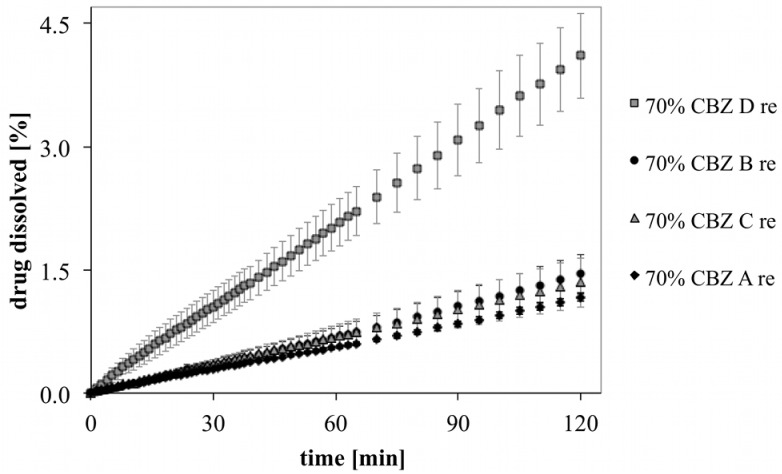
Initial drug release of recrystallized CBZ in binary mixtures with MCC.

Figure 9 shows the initial dissolution profiles of recrystallized CBZ in binary mixtures. The difference among recrystallized CBZ samples was reduced with the exception of the dissolution profiles of recrystallized CBZ D deviating significantly from the other dissolution profiles (*p* < 0.05). After 2 h dissolution, compacts showed swollen surfaces with wide creeks. This features were the strongest in MCC formulation with recrystallized CBZ D. It has to be considered that recrystallized CBZ samples were only gently broken apart by mortar and pistil and that particle size and shape was inhomogenous. Therefore, inhomogeneous mixture in MCC formulations cannot be excluded. 

## 4. Conclusions

Recrystallized CBZ samples presented a strongly reduced dissolution variability and the transformation to CBZ dihydrate was inhibited. Compacts of recrystallized CBZ samples also presented less variability in tensile strength compared to the untreated samples. The recrystallization did not induce any change in polymorphic form. Particle morphology of recrystallized CBZ samples was of irregular size and shape and may thus have resulted in inhomogeneous mixtures with MCC and to the deviation of one sample in the dissolution test. Therefore, more detailed studies are necessary to control the critical grinding step. The recrystallization in presence of 1% PVP can be suggested as a valid approach to control the dissolution variability in commercial CBZ samples. 
